# Primary Splenic Flexure Volvulus in a Young Adult With Cerebral Palsy: A Diagnostic and Management Dilemma

**DOI:** 10.7759/cureus.90654

**Published:** 2025-08-21

**Authors:** Mardiana Mardan, Dayang Corieza Febriany, Pradeep Chand Chandran, Mohamed Arif Hameed Sultan

**Affiliations:** 1 Department of Surgery, University Malaya Medical Centre, Kuala Lumpur, MYS; 2 Department of Radiology, Universiti Malaysia Sabah, Kota Kinabalu, MYS; 3 Department of Surgery, Hospital Queen Elizabeth, Kota Kinabalu, MYS; 4 Department of Surgery, Universiti Malaysia Sabah, Kota Kinabalu, MYS

**Keywords:** cerebral palsy, colonic volvulus, intestinal obstruction, splenic flexure volvulus, whirlpool sign

## Abstract

Colonic volvulus is an uncommon cause of intestinal obstruction. Among its subtypes, splenic flexure volvulus (SFV) is exceptionally rare, with only sporadic cases reported. It is most often associated with prior abdominal surgery or laxity of anatomical attachments. We describe a diagnostically challenging case of a 21-year-old man with spastic cerebral palsy and no history of abdominal surgery who presented with classical features of large bowel obstruction. CT imaging revealed a whirlpool sign at the splenic flexure, suggestive of volvulus. However, an emergency colonoscopy showed no evidence of torsion or mucosal ischemia. Because the patient was a poor surgical candidate, he was managed conservatively. Remarkably, his symptoms resolved, and he was discharged on day five of admission. Although rare, SFV should be considered in the differential diagnosis of acute abdomen, particularly in patients with neurodevelopmental disorders. Diagnostic uncertainty and delays in management may compromise patient outcomes. This case highlights a rare instance of spontaneous detorsion, underscoring the challenges of diagnosis, monitoring, and decision-making in a high-risk, noncommunicative patient, while also providing insights into radiographic findings, natural disease course, and recurrence risk.

## Introduction

Colonic volvulus refers to the torsion of a segment of the large intestine around its mesenteric axis, leading to bowel obstruction with potential progression to ischemia and necrosis. This phenomenon was first described by Von Rokitansky in the mid-nineteenth century. In Western populations, large bowel volvulus accounts for approximately 1-5% of intestinal obstruction cases, with the sigmoid colon (75-80%) and cecum (15%) being the most frequently involved segments [1,2]. In contrast, volvulus of the splenic flexure is exceptionally rare, comprising less than 2% of all colonic volvulus cases [3]. This rarity is attributed to the anatomical stability provided by the phrenicocolic, splenocolic, and gastrocolic ligaments that anchor the splenic flexure.

To date, only sporadic cases of splenic flexure volvulus (SFV) have been reported, with even fewer involving patients with neurodevelopmental conditions such as cerebral palsy [4,5]. In this population, chronic constipation, autonomic dysregulation, and altered bowel motility are thought to be contributing factors [4,6].

We report a diagnostically challenging case of SFV in a young adult with cerebral palsy and no history of prior abdominal surgery (“virgin abdomen”). This case is particularly notable for its spontaneous detorsion and successful conservative management, an outcome not widely documented in the literature. It underscores the diagnostic and clinical challenges encountered when managing large bowel obstruction in a medically vulnerable, nonverbal patient.

## Case presentation

A 21-year-old man from Kota Belud, Sabah, with a background of spastic cerebral palsy, was referred for evaluation of intestinal obstruction. He presented with a three-day history of postprandial vomiting, abdominal distension, and inability to pass stool or flatus. The patient was nonverbal and fully dependent for activities of daily living. His father, the sole caregiver and breadwinner, denied any prior episodes of constipation or abdominal complaints. The patient had not been attending special needs care due to financial limitations.

On examination, the patient was hemodynamically stable. The abdomen was markedly distended but soft, with sluggish bowel sounds and no signs of peritonism. Digital rectal examination revealed an empty rectum. Nasogastric decompression yielded 500 mL of gastric content. Laboratory investigations showed mild leukocytosis with normal serum electrolytes, arterial blood gases, and lactate levels.

**Figure 1:**
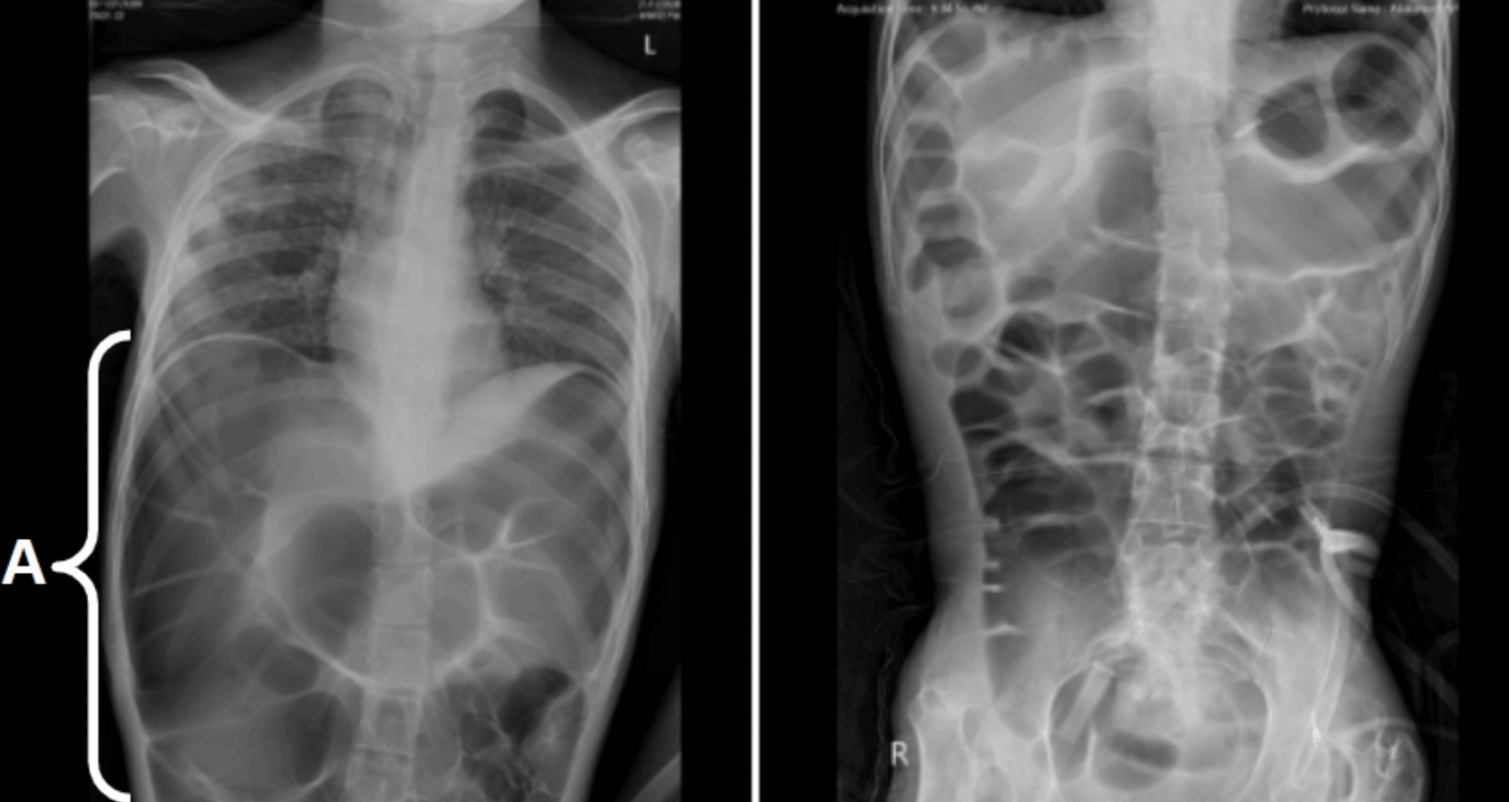
Erect chest radiograph (left) and abdominal radiograph (right) The erect chest radiograph shows dilated large bowel loops under both hemidiaphragms (marked by A), suggestive of proximal colonic obstruction. The abdominal radiograph demonstrates gross colonic dilatation without evidence of pneumoperitoneum.

A chest radiograph demonstrated dilated large bowel loops beneath both hemidiaphragms, while an abdominal radiograph further accentuated the dilated bowel loops (Figure 1).

**Figure 2:**
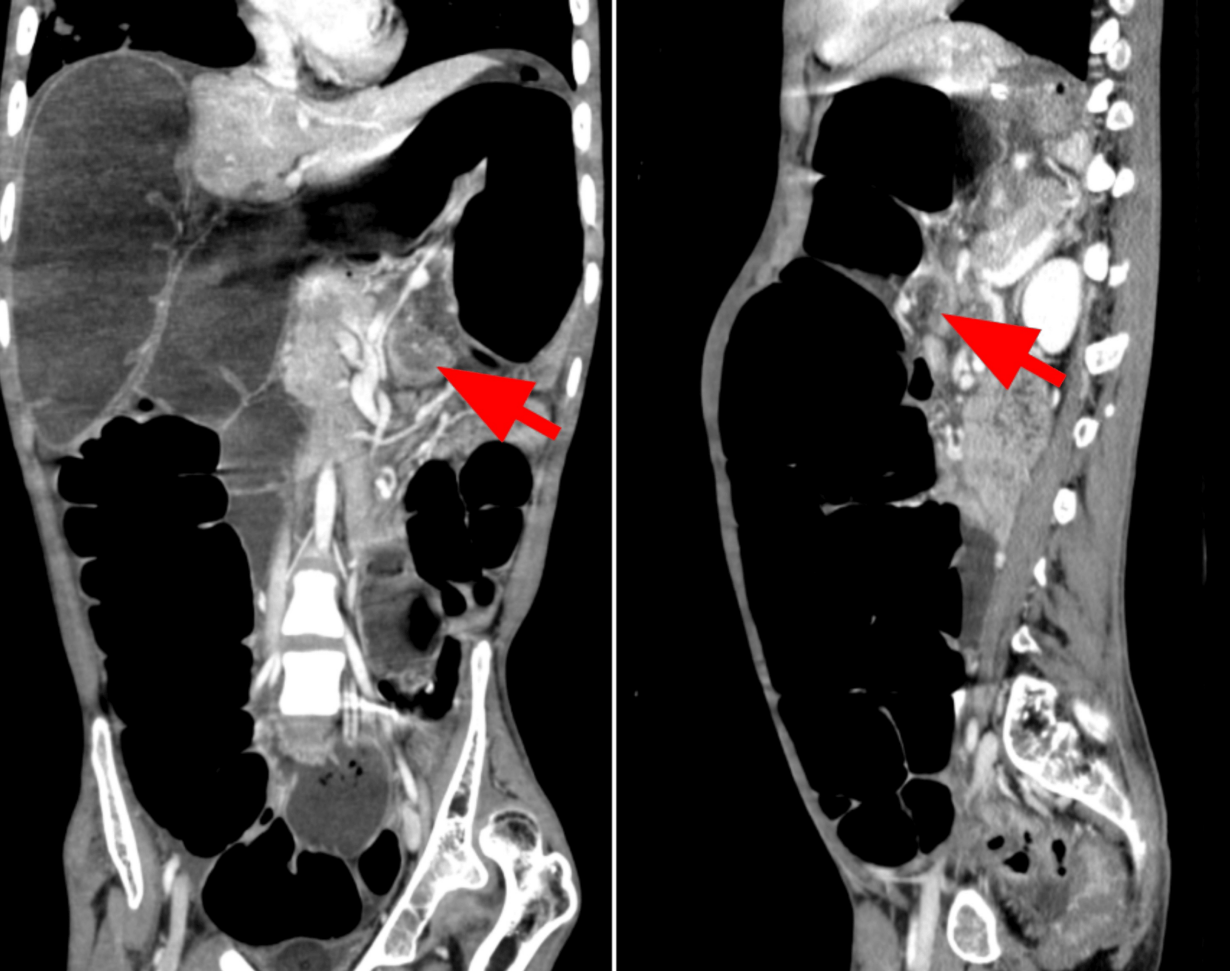
Contrast-enhanced CT of the abdomen and pelvis in coronal (left) and sagittal (right) projections The images show a grossly dilated right hemicolon and redundant transverse colon. A whirlpool sign (red arrow) is visible at the splenic flexure along the AP axis, indicating the transition point. Distally, the descending and sigmoid colon appear collapsed. These CT features confirm bowel obstruction secondary to SFV. SFV, splenic flexure volvulus

Contrast-enhanced CT of the abdomen and pelvis (Figure 2) revealed grossly dilated cecal, ascending, and redundant transverse colon (up to 7.7 cm), with a collapsed distal colon. A classic whirlpool sign was seen at the splenic flexure in the AP axis, indicating the site of torsion. No pneumoperitoneum or bowel ischemia was noted.

An emergency colonoscopy was performed. The colonoscope was advanced to the ascending colon without encountering a definitive point of torsion. No mucosal ischemia or obstructing lesions were noted. A flatus tube was inserted, and the patient was admitted for close observation.

Over the next 48 hours, the patient’s symptoms resolved. Bowel sounds returned, abdominal distension improved, and he resumed oral intake. He was discharged on day 5 of admission. Upon discharge, his father was advised on maintaining adequate hydration, increasing fiber intake, and monitoring for red flag symptoms. Outpatient follow-up was arranged in the surgical and developmental pediatric clinics, and social services were engaged to provide ongoing support for the family.

At the one-month follow-up in the outpatient surgical clinic, the patient remained clinically well, with no recurrence of symptoms or complications.

## Discussion

Volvulus is characterized by the rotation of a bowel segment around its mesentery, resulting in narrowing and obstruction of both ends at the point of torsion. This condition was first described in the mid-nineteenth century by Von Rokitansky. In Western populations, large bowel volvulus accounts for up to 5% of intestinal obstruction cases. The most common sites are the sigmoid colon (80%) and cecum (15%), segments of bowel not attached to the retroperitoneum [1,2]. In contrast, the splenic flexure is stabilized by ligamentous attachments to the stomach (gastrocolic), spleen (splenocolic), and diaphragm (phrenicocolic), making volvulus at this site unusual and accounting for only 2% of all colonic volvulus cases [3]. This rarity makes the condition clinically challenging to diagnose and prone to delays in treatment.

SFV can be categorized as primary or secondary. Primary SFV is associated with congenital anomalies such as absence or laxity of the splenic ligaments or a wandering spleen. Secondary SFV, which accounts for most cases, usually arises from acquired factors such as ligamentous disruption, mobilization of the splenic flexure, or adhesions from prior surgery, particularly operations involving the left colon.

The risk factors for SFV are multifactorial and often overlap with these etiologies. In individuals with congenital abnormalities, chronic constipation is the main risk factor, followed by conditions such as cerebral palsy, myotonic dystrophy [4,5], and Hirschsprung disease [6]. In patients with a history of abdominal surgery, particularly involving the splenic flexure, postoperative adhesions may predispose to secondary SFV.

The clinical presentation of SFV typically mirrors that of large bowel obstruction. Common symptoms include abdominal pain, vomiting, constipation, and inability to pass stool or flatus. Some patients may also present with blood-stained or mucoid rectal discharge. On examination, findings may include abdominal distension, tenderness, and a palpable mass. Bowel sounds are often initially hyperactive but diminish over time. In delayed presentations, or when diagnosis is uncertain, systemic toxicity may develop, manifesting as hypotension, fever, and altered mental status, suggesting bowel ischemia, necrosis, or perforation with evolving peritonitis and sepsis [4-6].

Diagnosis relies on clinical assessment supported by imaging. Abdominal radiographs may reveal suggestive but nonspecific findings, such as a markedly dilated air-filled colon with abrupt tapering at the splenic flexure [3,4]. Although rarely performed today, a barium enema can demonstrate the classic bird’s beak appearance at the torsion site. Cross-sectional imaging, particularly contrast-enhanced CT, is the most informative modality [5]. In our case, CT demonstrated a grossly dilated proximal colon and the classic “whirlpool sign” at the splenic flexure, representing twisted mesenteric vessels. This hallmark finding confirmed the diagnosis, identified the level of obstruction, and excluded ischemia or perforation. Such imaging is especially critical in patients with neurodevelopmental disorders, where communication limitations may delay recognition of abdominal pathology.

Our patient’s spastic cerebral palsy likely contributed to colonic dysmotility and chronic distension, predisposing him to volvulus. Neurogenic bowel dysfunction is well recognized in neurologic disorders, and colonic motor disturbances are implicated in chronic and slow-transit constipation [7]. Clinically, the patient showed symptoms of obstruction, but radiographically, SFV posed a diagnostic challenge. The classic “coffee bean sign” of sigmoid volvulus was absent, likely due to the redundant transverse colon and AP axis of torsion, which can obscure plain-film features. CT was crucial in identifying the obstruction, marked proximal dilation, and the pathognomonic whirlpool sign. The absence of ischemia or perforation allowed for conservative management with endoscopic decompression and close observation.

Several published reports offer comparisons. Hsueh et al. (2007) [5] described a 15-year-old boy with developmental delay who presented with abdominal distension and vomiting; imaging revealed classic SFV features, and the patient underwent laparotomy with colopexy, recovering postoperatively. Mittal et al. (2007) [3] reported a 24-year-old man with post-polio paralysis who presented with obstruction and fever; imaging demonstrated the coffee bean sign, and resection with end colostomy was required. In the UK, Umbu et al. (2022) [6] described a 40-year-old man with a prior colectomy who developed SFV; CT confirmed volvulus, and surgical resection was performed successfully. Bencic et al. (2022) [4] reported a 42-year-old man with cerebral palsy, epilepsy, and tetraplegia in whom CT revealed a whirl sign; the patient was successfully managed with colonoscopic decompression. Compared to these cases, our patient, a 21-year-old man with spastic cerebral palsy and no surgical history, presented with classical obstructive symptoms and a CT whirlpool sign. However, colonoscopy revealed no mucosal ischemia or fixed torsion, and he experienced spontaneous detorsion with complete recovery under conservative management. This case is notable for the absence of prior surgery (suggesting a primary anatomical basis) and for the rare occurrence of spontaneous resolution. It reinforces the diagnostic value of CT when classic radiographic signs are absent and highlights the need for vigilance in neurologically impaired, nonverbal patients.

The top priority in bowel obstruction is resuscitation, including fluid replacement, nil per os, and early antibiotics to minimize bacterial translocation and systemic compromise [8]. Although WSES guidelines primarily address sigmoid volvulus, these principles apply to all large bowel volvulus, including SFV [8]. Subsequent management includes decompression, resection, or colopexy [9]. Decision-making depends on hemodynamic stability, peritonitis, and overall status. In stable patients without peritonism, endoscopic decompression may be attempted [5,6]. If reduction fails or ischemia is present, urgent surgery is indicated. Resection of the splenic flexure with primary anastomosis (with or without diverting ileostomy or end colostomy) may be performed based on patient factors. In high-risk or frail patients, nonresection colopexy can prevent recurrence by anchoring the colon using sutures, Gore-Tex strips, or extraperitonealization [5]. Although conservative strategies have been described, surgical resection remains the definitive treatment due to high recurrence risk [4,6].

Rarely, spontaneous detorsion has been reported before surgical intervention [6,10,11]. Spontaneous resolution occurs in approximately 2% of sigmoid volvulus cases [9], with some series reporting rates as low as 0.6% [10]. In our case, symptom resolution, improved abdominal signs, and radiographic decompression over 48 hours confirmed detorsion. Although colonoscopy did not show torsion, we acknowledge that insufflation may have contributed to the reduction, which was considered retrospectively after clinical improvement. Nonetheless, recurrence remains a concern. Patients require close monitoring, given the risk of ischemic or necrotic bowel. Literature reports high recurrence after nonoperative management; in sigmoid volvulus, recurrence rates following endoscopic decompression range from 15% to 55% [9], supporting elective fixation or resection in recurrent cases [4,6].

Once discharged, close follow-up is essential. Caregivers should be educated on red flag symptoms and the need for urgent evaluation if they occur. In this case, coordination with social services and developmental pediatric support ensured ongoing monitoring. The patient’s father (primary caregiver) was specifically counseled to watch for progressive abdominal distension, vomiting, and constipation. Empowering caregivers to recognize early recurrence signs is critical in nonverbal or dependent patients, where delays may result in catastrophic outcomes.

At one-month follow-up, the patient remained asymptomatic with no evidence of recurrence.

This case highlights several key points: (1) Although rare, SFV should be considered in patients with chronic constipation and neurodevelopmental conditions such as cerebral palsy; (2) Early recognition and prompt resuscitation are crucial, as delays may lead to ischemia or perforation; (3) Abdominal radiographs may be nonspecific, making CT vital for diagnosis; and (4) Definitive surgical management remains the standard, with resection and primary anastomosis preferred when feasible.

## Conclusions

SFV is an exceptionally rare form of colonic volvulus, often associated with chronic constipation or prior abdominal surgery. Its rarity and nonspecific presentation create significant diagnostic challenges, particularly in patients with communication barriers or neurodevelopmental conditions. Delayed diagnosis can result in life-threatening complications, including bowel ischemia, perforation, sepsis, multiorgan failure, and death.

Cross-sectional imaging, particularly CT with identification of the whirlpool sign, plays a pivotal role in timely and accurate diagnosis. Although surgical resection remains the definitive treatment to prevent recurrence, our case was unusual in that the patient had no history of abdominal surgery and experienced spontaneous detorsion with successful conservative management. This underscores the importance of considering SFV even in a “virgin abdomen,” especially in neurologically impaired patients. Ultimately, this case highlights the need for high clinical vigilance, the utility of advanced imaging, and individualized decision-making when managing rare causes of bowel obstruction. Equally important is empowering caregivers through education about red flag symptoms and ensuring close follow-up, particularly for nonverbal or dependent individuals.
